# Comparative Results of “Epi-Off” Conventional versus “Epi-Off” Accelerated Cross-Linking Procedure at 5-year Follow-Up

**DOI:** 10.1155/2020/4745101

**Published:** 2020-07-22

**Authors:** Cristina Ariadna Nicula, Dorin Nicula, Anca Maria Rednik, Adriana Elena Bulboacă

**Affiliations:** ^1^Department of Ophthalmology, Iuliu Hațieganu University of Medicine and Pharmacy, Cluj-Napoca, Romania; ^2^Oculens Clinic, Cluj-Napoca, Romania; ^3^County Eye Hospital, Cluj-Napoca, Romania; ^4^Department of Physiopathology, Iuliu Hațieganu University of Medicine and Pharmacy, Cluj-Napoca, Romania

## Abstract

**Purpose:**

The aim of our study was to compare the long-term efficacy and safety of “epi-off” conventional and “epi-off” accelerated corneal cross-linking (CXL) in patients with progressive keratoconus.

**Methods:**

“Epithelial-off” (“Epi-off”) CXL using the conventional technique (3 mW/cm^2^, 30 minutes) was performed in 93 eyes of 93 patients (S-CXL group) and “epi-off” accelerated method (9 mW/cm^2^, 10 minutes) in 76 eyes of 76 patients with progressive KCN (A-CXL group). Cases with different stages of keratoconus and topographic evidence of progression were included. Main outcomes comprised refraction, keratometry measurements, uncorrected (UCVA) and best-corrected visual acuity (BCVA), and topographical indices. Micromorphological analysis was assessed by anterior segment ocular coherence tomography (AS-OCT). The follow-up period was 5 years.

**Results:**

In both groups, Kflat presented similar results: decrease at 1 year (*p*=0.465), at 2 years (*p*=0.672), at 3 years (*p*=0.198), at 4 years (*p*=0.32), and at 5 years (*p*=0.864). In both groups, Ksteep presented a similar decrease at 1 year (*p*=0.709), at 2 years (*p*=0.455), at 3 years (*p*=0.43), at 4 years (*p*=0.57), and at 5 years (*p*=0.494), with no statistically significant difference. Decrease in Kavg was similar in both groups at all analyzed time points (*p*=0.18 at 1 year, *p*=0.093 at 2 years, *p*=0.57 at 3 years, *p*=0.154 at 4 years, and *p*=0.247 at 5 years). Kmax had a similar decrease in both groups at 1 year (*p*=0.06), at 2 years (*p*=0.09), at 3 years (*p*=0.126), at 4 years (*p*=0.113), and at 5 years (*p*=0.114). There was no statistically significant difference between the cylinder decrease in both groups (*p*=0.349 at 1 year, *p*=0.6782 at 2 years, *p*=0.299 at 3 years, *p*=0.0943 at 4 years, and *p*=0.144 at 5 years). The BCVA values were statistically significantly higher than the preoperative values in both groups at all time points (*p* < 0.05). Topographical indices such as thinnest corneal point (TP), corneal volume (CV), index vertical asymmetry (IVA), index of vertical asymmetry (ISV), index of height asymmetry (IHA), index of height decentration (IHD), Belin/Ambrosio Enhanced Ectasia Display (BAD_D), and Ambrosio retinal thickness (ART Max) were significantly statistically decreased compared with baseline at all time points, in both groups.

**Conclusion:**

“Epi-off” accelerated and conventional CXL have the same efficacy in terms of improvement in visual and topographic outcomes.

## 1. Introduction

Keratoconus (KCN) is a bilateral ectatic corneal disorder, frequently asymmetric, with a progressive thinning of the cornea resulting in protrusion, progressive irregular astigmatism, and visual deterioration [1]. In the majority of cases, this condition affects young patients with an early age of onset as a negative prognostic factor for evolution and corneal transplantation [2]. That is why, an early detection of KCN by using the topographical and tomographical evaluation of the Pentacam device is of major importance [3, 4]. During the past years, corneal collagen cross-linking (CXL) became the standard procedure for KCN therapy. CXL has been used in dentistry, orthopedics, and dermatology for many years, and since 1998, it has been tested and subsequently introduced in the treatment of KCN together with riboflavin (a nontoxic photosensitizing agent) and ultraviolet irradiation (UVA) [5]. The final effect of the CXL technique is represented by the strengthening of the cornea, and the goal is to slow down or stop the progression of KCN, thus avoiding or delaying the necessity of keratoplasty.

CXL technique consists in the photopolymerization of the stromal fibrillar tissue, in order to increase their stiffness and resistance to corneal ectasia and proteolytic enzymes (collagenase) [6, 7], reducing corneal permeability [8] and formation of large collagen molecular aggregates [9] through the combined action of the photosensitizing substance (riboflavin—B2) and ultraviolet A (UVA) light irradiation performed with an illuminator in a solid state of UVA kind [5, 9]. The conventional (standard) technique (“epi-off” technique) named as the Dresden protocol uses riboflavin which is exposed to a measured dose of long wavelength UVA radiation (370 nm) at 3mw/cm^2^ for 30 minutes, applied after epithelial removal and resulting in a total energy dose of 3.47 or a radiant exposure of 5.47 cm [2, 9, 10]. Several studies demonstrated the safety and long-term efficacy of the conventional “epi-off” cross-linking in stabilizing progressive KCN [11–19].

In order to reduce the time of treatment and the patient discomfort and to avoid the excessive corneal dehydration and thinning that can occur for a period of 30 minutes, the accelerated “epi-off” CXL was introduced. By applying a higher intensity (9 mw/cm^2^) for a shorter period of time (10 minutes), the same level of radiant exposure as in the conventional CXL can be achieved [20, 21]. This is according to Bunsen–Roscoe's law of reciprocity that showed that an increase of UVA irradiation associated with a reduced exposure time theoretically delivers a total energy dose to the tissue similar to that in the conventional treatment, with the same biological effect [20, 21]. Previous ex vivo experiments on porcine corneas performed with high energy and short irradiation time settings have revealed similar results on the biomechanical properties compared to the standard protocol [20]. The potential advantages of the accelerated CXL technique include decreased exposure time, improved comfort of the patient, and inferior infection risk [22]. Furthermore, there are studies that demonstrated the efficiency of “epi-off”accelerated CXL [20, 23–25]. However, Hashemi et al. [26] compared the two-year changes in dynamic corneal response evaluated by Corvis ST, between 18 mW/cm^2^ (5 minutes) and 9 mW/cm^2^(10 minutes) CXL protocols. The conclusion was that both procedures increased corneal stiffness, especially the 9 mW/cm^2^ procedure. Moreover, there are studies that compared the outcomes of “epi-off” conventional and accelerated CXL procedures and showed comparable effect in preserving keratometry parameters in both techniques [22, 27–33]. Furthermore, Rechichi et al. [34], in a prospective, multicenter, and interventional study, evaluated intraoperative corneal pachymetry in patients undergoing pulsed-light accelerated CXL with dextran-free hydroxyl-propyl methylcellulose 0.1% riboflavin solution (8 minutes of exposure, 30 mW/cm^2^, and an energy dose of 7.2 J/cm^2^) and revealed no statistically significant intraoperative corneal thickness reduction.

Evaluation of the demarcation line is considered as a measurement of the depth of CXL treatment into the stroma [35]. Corneas that underwent CXL treatment present an inferior rate of enzymatic collagenase degradation when compared to an untreated cornea [35]. Some studies [36, 37, 39] showed the role of the demarcation line after CXL depth as representative of CXL effectiveness.

The aim of our study was to assess the long-term efficacy and safety of “epi-off” conventional and accelerated CXL by comparing the keratometry measurements, spherical and cylinder equivalents, visual acuity, and topographical indices in patients with progressive keratoconus up to 5 years, following the procedure, and also to determine the qualitative corneal changes evaluated by anterior segment optical coherence tomography (AS-OCT) after both procedures.

## 2. Materials and Methods

A retrospective single-center study was performed at the Oculens Private Clinic in Cluj-Napoca, Romania, after the unanimous approval of the clinic ethics committee (5/2020) and adhered to the tenets of Helsinki Declaration (1964). Two groups of patients were included in our study. The conventional group included 93 eyes of 93 patients, with progressive KCN who underwent CXL by the conventional (standard) “epi-off” technique (S-CXL group). The accelerated group included 76 eyes of 76 patients, with progressive KCN who underwent CXL by the accelerated “epi-off” procedure (A-CXL group). All patients included in the study were affected by KCN with a documented clinical and instrumental worsening at least in the last three to six months of observation: myopia and astigmatism changes >3D, a mean change of central K value > 1.5D in three consecutive corneal topographic measurements, increase in the maximum keratometry (Kmax) in topography of more than 1D, or a mean decrease in central corneal thickness >5% in three consecutive tomographic measurements [39]. The “epi-off” conventional CXL was performed between January 2011 and January 2013 and the “epi-off” accelerated CXL between February 2013 and January 2015. All cases were followed up postoperatively at 1 month, 6 months, and 1, 2, 3, 4, and 5 years, following the procedure.

The inclusion criteria for both groups included age greater than 18 years, any gender, progressive KCN of different stages (according to the Amsler–Krumeich classification), and an average corneal thickness of at least 400 *µ*m at the thinnest corneal location. Patients with previous intracorneal ring placement, corneal pachymetry less than 400 *µ*m, Vogt striae presence, history of herpetic keratitis or other corneal infections, dry eye syndrome, aphakia, central corneal scar, autoimmune illnesses, nystagmus, and pregnancy were excluded.

Before the procedure, a complete ocular assessment was performed, including uncorrected (UCVA) and best-corrected visual acuity (BCVA), refractometry (manifest and cycloplegic), keratometry-steep (Ksteep), flat (Kflat), average (Kavg), and maximum (Kmax) (Topcon auto refracto-keratometer, KR 8900, Japan), slit-lamp exam (Slit Lamp BX 900, Haag-Streit AG3098 Koeniz, Switzerland), eye fundus examination, intraocular pressure measured by applanation tonometry, corneal tomography (Pentacam® HR Premium; Oculus Optikgerate GmbH, Wetzlar, Germany), and endothelial cell counting (Konan SP-9000, Hyogo, Japan). Visual acuity was measured with the Snellen charts. For scientific reasons, it was transformed as the logarithm of minimum angle of resolution (LogMar). Patients were requested to discontinue the wear of contact lens 2 weeks before evaluation or surgery.

After signing the specific informed consent, the two groups of patients underwent the “epi-off” conventional or “epi-off” accelerated CXL procedure.

The CXL procedure was performed in the operating room in sterile conditions. The “epi-off” CXL technique was used for both conventional and accelerated procedures. During the procedure, a single 3.0 ml of riboflavin 0.1%-dextran 20% solution (Peschke D) was opened, and the power of the UVA illuminator (Peschke Meditrade GmbH, Huenenberg, Switzerland CXL system) was verified. Before the procedure, one drop of Isopto® Carpine (Amman Pharma, Romania) was instilled in the eye and was prepared with topical anesthesia with oxybuprocaine hydrochloride 0.4% (Benoxi, Unimed Pharma Ltd), 1-2 drops, 10–15 minutes prior to cross-linking was instilled. Sterile operating field and lid speculum were used. A corneal de-epithelization with a blunt spatula on a 9 mm diameter optical zone was performed, followed by the instillation of riboflavin 0.1%-dextran 20% solution (Peschke® D, Huenenberg, Switzerland), every 2 minutes for 30 minutes before irradiation. Pachymetry was done after epithelial removal to ascertain that the corneal depth was more than 400 m. The optical corneal zone was additionally aligned under a UVA power of 3 mW/cm^2^ for 30 minutes in conventional CXL (Dresden protocol) (total energy:7.2 J/cm^2^) and under a power of 9 mW/cm^2^ for 10 minutes in accelerated CXL (total energy:5,4 J/cm^2^). Riboflavin 0.1% every 2 minutes was instilled during irradiation in both groups. The cornea surface was irrigated with balanced salt solution after irradiation. At the end of the surgery, steroids and antibiotics (Tobradex) (Alcon Novartis, Dallas, Worth, USA) were instilled. A bandage soft contact lens was applied for 3 days until the corneal healing was completed. After the procedure, all patients received topical antibiotics and steroids such as Tobradex (Alcon Novartis, Dallas, Worth, USA) 5 times/day, for one month, and artificial tears 2 times/day for 2 to 3 months. Both groups were followed up postoperatively in the clinic at day 1, day 3, month 1, month 6, year 1, and yearly up to 5 years. Ocular refraction, keratometry measurements, uncorrected visual acuity (UCVA), best-corrected visual acuity (BCVA), slit-lamp examination, corneal tomography, and topography were performed at all visits, except for the first postoperative day, 4 weeks, and 6 months. Corneal topographic and aberrometer parameters were recorded from all the examinations. At one month after the procedure (in both groups), anterior segment optical coherence tomography (AS-OCT) analysis (Triton OCT, Topcon Corporation, Tokyo, Japan) was done in order to assess qualitative A-CXL- and S-CXL-induced corneal changes and treatment penetration looking after demarcation line. The image was captured when the corneal reflex was visible, and the depth of demarcation line was measured using the caliper tool provided by the manufacturer.

### 2.1. Statistical Analysis

Data were presented using the number (percentage) for categorical variables and mean ± standard deviation or median (quartile 1; quartile 3) for continuous variables. Groups were compared using the independent Student *t*-test for continuous variables with normal distribution, the median test for continuous variables with abnormal distribution, the and chi-square test for categorical variables. The evolution of variables at different time points was compared with baseline values using the paired *t*-test. A *p* value <0.05 was considered as statistically significant.

## 3. Results

We analyzed data of 93 eyes of 93 patients with progressive KCN who were treated with the conventional (standard) “epi-off” CXL technique (S-CXL group) and 76 eyes of 76 patients treated with the “epi-off” accelerated CXL (A-CXL group). At baseline, the mean age was 26.5 ± 8.5 years (ranging between 18 and 54 years) in the S-CXL group and 29 ± 8.2 years (ranging between 18 and 48 years) in the A-CXL group (*p* value for the difference between groups = 0.064). There were 52 males (55.9%) in the S-CXL group and 41 males (53.9.3%) in the A-CXL group (*p*=0.184). Regarding the stage of keratoconus (Amsler–Krumeich staging), 8.6% had stage I, 53.76% of patients had stage II, and 37.63% stage III in the S-CXL group. In the A-CXL group, 7.89% had stage I, 47.37% of patients had stage II, and 44.74% stage III (Table 1).

### 3.1. Corneal Findings during the Treatment

**Figure 1:**
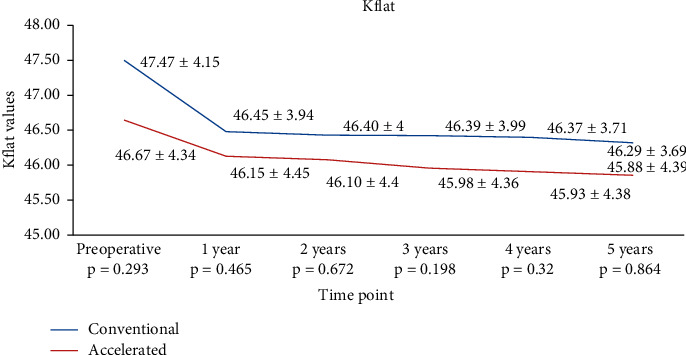
Evolution of Kflat in both groups.

There was no statistically significant difference between study groups in terms of preoperative flat keratometry (Kflat) (*p*=0.293), steep keratometry (Ksteep) (*p*=0.098), mean keratometry (Kavg) (*p*=0.309), and Kmax (*p*=0.179). In the S-CXL group, Kflat dropped from 47.47 ± 4.15 D to 46.45 ± 3.94 D at 1 year (*p*=0.037), to 46.4 ± 4.00 D (*p*=0.025) at 2 years, to 46.39 ± 3.99 D (*p*=0.0098) at 3 years, to 46.37 ± 3.71 D at 4 years (*p*=0.0046), and to 46.29 ± 3.69 D (*p*=0.0064) at 5 years. In the A-CXL group, Kflat dropped from 46.67 ± 4.34 D to 46.15 ± 4.45 D at 1 year (*p*=0.0433), to 46.1 ± 4.40 D at 2 years (*p*=0.0049), to 45.98 ± 4.36 D at 3 years (*p*=0.039), to 45.93 ± 4.38 D at 4 years (*p*=0.0066), and to 45.88 ± 4.39 D (*p*=0.0039) at 5 years. Kflat presented a similar decrease in both groups at 1 year (*p*=0.465), at 2 years (*p*=0.672), at 3 years (*p*=0.198), at 4 years (*p*=0.32), and at 5 years (*p*=0.864) (Table 2 and Figure 1).

**Figure 2:**
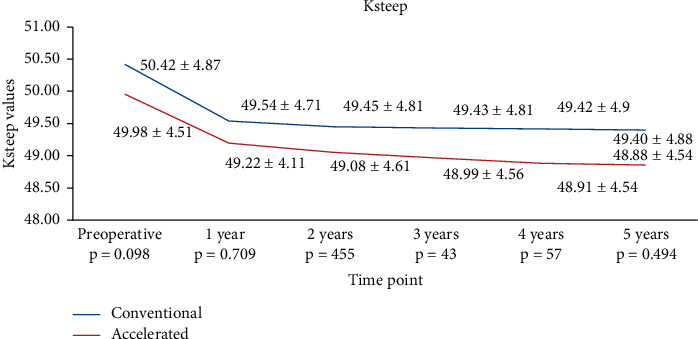
Evolution of Ksteep in both groups.

In the S-CXL group, Ksteep dropped from 50.42 ± 4.87 D to 49.54 ± 4.71 D at 1 year (*p*=0.0043), to 49.45 ± 4.81 D (*p*=0.0023) at 2 years, to 49.43 ± 4.81 D (*p* < 0.0001) at 3 years, to 49.42 ± 4.90 D at 4 years (*p*=0.0012), and to 49.40 ± 4.88 D (*p*=0.0008) at 5 years. In the A-CXL group, Ksteep dropped from 49.98 ± 4.51 D to 49.22 ± 4.11 D at 1 year (*p*=0.0072), to 49.08 ± 4.61 D at 2 years (*p*=0.0009), to 48.99 ± 4.56 D at 3 years (*p*=0.027), to 48.91 ± 4.54 D at 4 years (*p*=0.0036), and to 48.88 ± 4.54 D at 5 years (*p*=0.002) (Table 2 and Figure 1). Ksteep presented a similar decrease in both groups at 1 year (*p*=0.709), at 2 years (*p*=0.455), at 3 years (*p*=0.43), at 4 years (*p*=0.57), and at 5 years (*p*=0.494), with no statistically significant difference (Table 2 and Figure 2).

**Figure 3:**
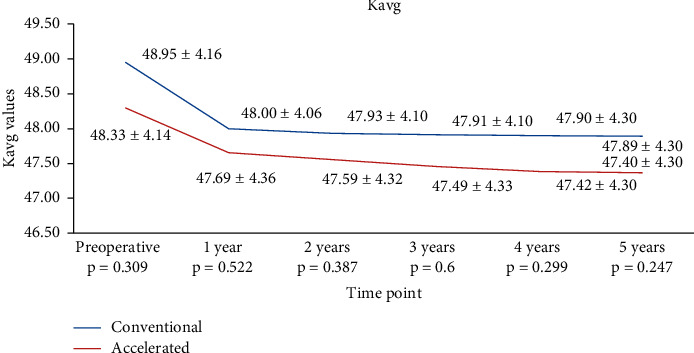
Evolution of Kavg in both groups.

For Kavg, the decrease was similar in both groups at all time points analyzed. In the S-CXL group, Kavg dropped from 48.95 ± 4.16 D to 48.00 ± 4.06 D (*p*=0.0194) at 1 year, to 47.93 ± 4.10 D (*p*=0.0067) at 2 years, to 47.91 ± 4.10 D (*p*=0.0074) at 3 years, to 47.90 ± 4.30 D (*p*=0.0211) at 4 years, and at to 47.89 ± 4.30 at 5 years (*p*=0.011). In the A-CXL group, Ksteep dropped from 48.33 ± 4.14 D to 47.69 ± 4.36 D at 1 year (*p*=0.01), to 47.59 ± 4.32 D (*p*=0.022) at 2 years, to 47.49 ± 4.33D (*p*=0.0068) at 3 years, to 47.42 ± 4.30 D (*p*=0.008) at 4 years, and to 47.40 ± 4.30 at 5 years (*p*=0.0053) (Table 2 and Figure 3). The reduction of Kavg from baseline data was 1.06D in the S-CXL group and 0.93D in the A-CXL group.

**Figure 4:**
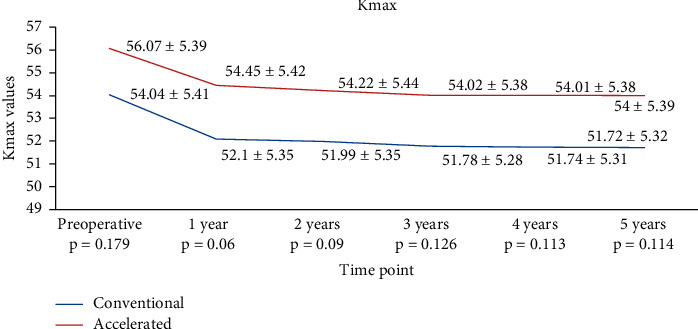
Evolution of Kmax in both groups.

For Kmax, the decrease was similar in both groups at all time points analyzed. In the S-CXL group, Kmax dropped from 54.05 ± 5.41 D to 52.10 ± 5.35 (*p*=0.0172) at 1 year, to 51.99 ± 5.35 D (*p*=0.012) at 2 years, to 51.78 ± 5.28 D (*p*=0.0086) at 3 years, to 51.74 ± 5.31 D (*p*=0.0075) at 4 years, and at to 51.72 ± 5.32 at 5 years (*p*=0.0071). In the A-CXL group, Kmax dropped from 56.07 ± 5.39 D to 54.45 ± 5.42 D at 1 year (*p*=0.0456), to 54.22 ± 5.44 D (*p*=0.0368) at 2 years, to 54.02 ± 5.38 D (*p*=0.0199) at 3 years, to 54.01 ± 5.38 D (*p*=0.0196) at 4 years, and to 54.00 ± 5.39 D at 5 years (*p*=0.0189) (Figure 4). There was a statistically significant decrease in Kmax in both groups comparing with baseline at all time points (Table 2). The reduction of Kmax from baseline data was 2.33 in the S-CXL group and 2.07 D in the A-CXL group.

Compared to preoperative status, for conventional and accelerated CXL, Kflat, Ksteep, Kavg, and Kmax were statistically significantly lower at year 1 and were maintained statistically significantly lower at all time points (*p* < 0.05 for all time points as compared to preoperative values) (Table 2).

**Figure 5:**
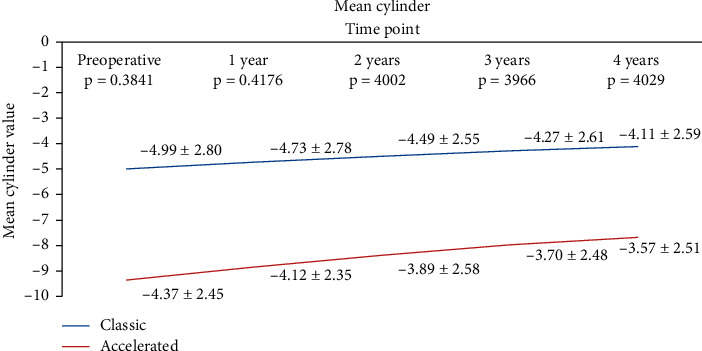
Evolution of cylinder in both groups.

In the S-CXL group, mean cylinder decreased from −4.415 ± 2.39 D to −3.905 ± 2.26 at 1 year (*p*=0.04), to −3.435 ± 2.22 D at 2 years (*p*=0.0093), to −3.37 ± 2.27 D at 3 years (*p*=0.0399), to −3.361 ± 2.26 D at 4 years (*p*=0.042), and to −3.358 ± 2.27 D at 5 years (*p*=0.033). In the A-CXL group, mean cylinder decreased from −4.15 ± 2.15 D to −3.661 ± 2.19 D at 1 year (*p*=0.033), to −3.105 ± 2.21 D at 2 years (*p*=0.028), to −3.076 ± 2.17 D at 3 years (*p*=0.0062), to −3.002 ± 2.18 D at 4 years (*p*=0.0309), and to −2.997 ± 2.22 at 5 years (*p*=0.022). There was a statistically significant difference in the cylinder value between baseline and all time point visits. There was no statistically significant difference between the decrease in the cylinder value between the two groups (*p*=0.349 at 1 year; *p*=0.6782 at 2 years; *p*=0.299 at 3 years; *p*=0.0943 at 4 years; *p*=0.081 at 5 years) (Figure 5).

**Figure 6:**
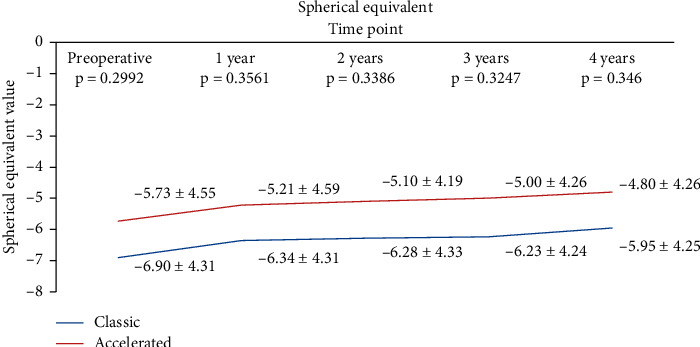
Evolution of SE in both groups.

Compared to baseline, in the S-CXL group, spherical equivalent (SE) decreased from −6.1 ± 4.2 D to −5.48 ± 3.93 D at 1 year (*p*=0.0065), to −5.1 ± 4.01 D at 2 years (*p*=0.0005), to −5 ± 4.12 D at 3 years (*p*=0.0128), to −4.92 ± 3.87 D at 4 years (*p*=0.0179), and to −4.9 ± 3.88 D at 5 years (*p*=0.0166). In the A-CXL group, SE decreased from baseline −5.89 ± 4 D to −5.27 ± 4.07 D at 1 year (*p*=0.0007), to −5.02 ± 3.97 D at 2 years (*p*=0.0081), to −4.87 ± 3.79 D at 3 years (*p*=0.004), to −4.82 ± 4.1 D at 4 years (*p*=0.01), and to −4.79 ± 4.1 D at 5 years (*p*=0.0109), with no statistically significant difference between groups at any time point (*p*=0.2119 at 1 year; *p*=0.92 at 2 years; *p*=0.4803 at 3 years; *p*=0.1866 at 4 years; *p*=0.087 at 5 years) (Figure 6).

**Figure 7:**
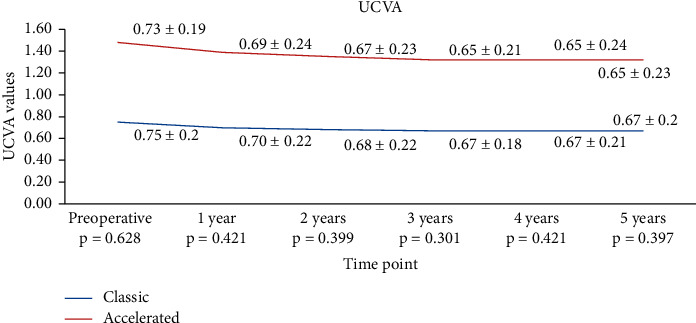
Evolution of UCVA in both groups.

There was no statistically significant difference in UCVA and BCVA between conventional and accelerated CXL in comparison with baseline values (*p*=0.6283/*p*=0.543). The preoperative UCVA in the S-CXL group was 0.75 ± 0.2 LogMar and remained 0.7 ± 0.22 LogMar at 1 year. At 2 years, UCVA increased to 0.68 ± 0.22 LogMar, to 0.67 ± 0.18 LogMar at 3 years, to 0.67 ± 0.21 LogMar at 4 years, and to 0.67 ± 0.20 at 5 years. The preoperative UCVA in the A-CXL group was 0.73 ± 0.19 LogMar and increased to 0.69 ± 0.24 LogMar at 1 year, to 0.67 ± 0.23 LogMar at 2 years, to 0.65 ± 0.21 LogMar at 3 years, to 0.65 ± 0.24 LogMar at 4 years, and became 0.65 ± 0.23 LogMar at 5 years. For BCVA, the improvement was not statistically significantly different between the A-CXL group and the S-CXL group at 1 year (*p*=0.1142), at 2 years (*p*=0.908), at 3 years (*p*=0.346), at 4 years (*p*=0.4575), and at 5 years (*p*=0.4072). Compared to baseline, in the S-CXL group, postoperative UCVA was statistically significantly lower than preoperative values at all time points analyzed (*p* < 0.001 for all time points). In the A-CXL group, postoperative UCVA was statistically significantly higher compared to baseline starting from year 1 and maintained higher than preoperative levels at years 3, 4, and 5 (*p* < 0.001) (Figure 7).

**Figure 8:**
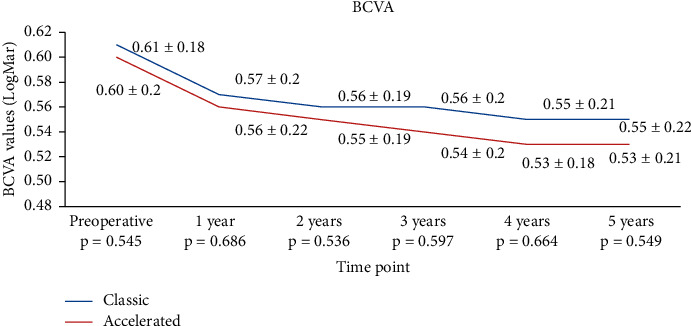
Evolution of BCVA in both groups.

For BCVA, the values were statistically significantly higher than the baseline values in both groups at all time points (*p* < 0.05) (Figure 8).

### 3.2. Topographical Indices

Thinnest corneal point (TP), corneal volume (CV), index vertical asymmetry (IVA), index of vertical asymmetry (ISV), index of height asymmetry (IHA), index of height decentration (IHD), Belin/Ambrosio Enhanced Ectasia Display (BAD_D), and Ambrosio retinal thickness (ART Max) were significantly statistically decreased compared with baseline at all time points in both groups (Table 3). We did not find any significantly statistically difference in the above parameters between S-CXL and A-CXL groups (*p* > 0.05) (Table 3).

The changes in corneal total high ocular aberration (HOA) and root mean square values (RMS) compared with the baseline decreased significantly and statistically in both groups (*p* < 0.05 and *p* < 0.005, respectively) but did not differ significantly between S-CXL and A-CXL groups (Table 4).

There was no statistically significant difference in the corneal stromal demarcation line depth between the two groups, with a mean depth of 214 ± 12.82 *μ*m in the S-CXL group and 203 ± 12.03 *μ*m in the A-CXL group (*p*=0.0736).

None of our patients in none of the groups lost lines of BCVA. In each case in both groups, complete epithelization was accomplished in 3 days. Infections or melting were not noticed in any of the studied group cases. Haze was present in the majority of cases in both groups for about 3 to 6 months but decreased progressively after this period.

## 4. Discussion

With recent modifications of the original Dresden protocol, accelerated CXL has become one of the interesting topics in corneal surgery. It has been shown that “epi-off” standard CXL (Dresden protocol) leads to stabilization of KC, with flattening of topographic keratometry and improvement of visual acuity in many cases [19, 40–44].

In our study, we aimed to evaluate the evolution of patients up to 5 years, following “epi-off” standard and accelerated CXL procedures. Results of our study showed a similar reduction in Kavg, Ksteep, and Kflat in both accelerated and conventional study groups at all time points (*p* < 0.05), maintained at 5 years. In a comparative study of accelerated (30 mW/cm^2^ for 3 minutes at 5.4 J/cm^2^) versus conventional CXL (Dresden protocol), Tomita et al. [30] reported for the first time a significant flattening of keratometry measurements in both groups at 1 year, following the procedure, with no statistically significant difference between the study groups (ΔK = −0.62 D in the accelerated group and ΔK = −1.77 D in the conventional group, *p*=0.21). They also showed that the difference between the mean demarcation line depth in both groups was not statistically significant (294.38 ± 60.57 *μ*m in the accelerated group and 380.78 ± 54.99 *μ*m in the conventional group). Furthermore, in a report which compared the results of accelerated (7 mW/cm^2^ irradiation 15 minutes protocol) versus conventional CXL (Dresden protocol), Kanellopoulos [45] reported the flattening of steep keratometry and the stabilization of KCN in both groups (from 49.5 D to 46.1 D in the accelerated group and from 48.7 D to 45.8 D in the conventional group). Similarly, Shetty et al. [46] revealed that the accelerated CXL (9 mW/cm^2^ for 10 minutes and 18 mW/cm^2^ for 5 minutes) had comparable outcomes to standard CXL, but the accelerated CXL using 30 mW/cm^2^ for 3 minutes was not as efficient. In another study, in which 77 eyes treated with accelerated CXL and 76 treated with conventional CXL were enrolled and followed for 15 months, Hashemian et al. [47] showed a similar decrease in Ksteep at 15 months in both study groups (ΔKsteep = −1.98 D in the conventional group and ΔK = −1.85 D in the accelerated group, *p*=0.36). Several other studies [22, 29, 31, 48] showed comparable successful clinical results regarding the two procedures. Moreover, Yildirim et al. [49] had published the results of a study which compared two different types of accelerated CXL (30 mw/cm^2^ for 4 minutes and 18 mW/cm^2^ for 5 minutes) and revealed no statistically significant changes in topographical and corneal measurements between the two groups. Furthermore, Mazzota et al. [50] demonstrated better functional results and deeper stromal penetration in pulsed-light accelerated CXL compared to continuous light accelerated CXL treatment with a follow-up of one year. Scherif [51] compared the Dresden protocol with the accelerated CXL (30 mW/cm^2^ 4 minutes 20 seconds) and demonstrated that there was no statistically significant difference between the two groups in terms of clinical results. Conflicting studies showed that conventional CXL improved BCVA and decreased Kmax and Kmean, but the accelerated procedure provided unchanged BCVA, Kmax, and Kmean [52]. Moreover, Choi et al. [53] and Peyman et al. [54] revealed that an accelerated KCL procedure of 30 mW/cm^2^ 3 minutes 40 seconds provided a lower efficiency compared with the Dresden protocol, demonstrated by the depth of the demarcation line. It was postulated that the reduced effect of 30 mW/cm^2^ in 3-4 minutes is linked with the decrease in oxygen in these high-fluence treatments [55]. The introduction of pulse treatments aimed to restore oxygen in the cornea [55].

Our findings showed a statistically significant reduction in Kmax compared to baseline in both groups (*p* < 0.05) which changed during the 5 years of follow-up. We used this parameter not only to establish the progression of keratoconus, but also as an indicator of successful results in CXL. Similarly, Kirgiz et al. [32] reported in a comparative study between two procedures of accelerated CXL (18 mW/cm^2^ in 5 minutes and 9 mW/cm^2^ in 10 minutes) that Kmax is a useful index of CXL evaluation results, with a statistically significant decrease in accelerated CXL in 10 minutes.

We observed a decrease in both cylinder and spherical equivalents after CXL in our samples, with no statistically significant difference in the change between the study groups. Our results are similar with those reported by Woo et al. [22] who enrolled 76 patients in a prospective study and reported no difference between accelerated and standard CXL beyond the 3 months after surgery. Hashemian et al. [28] and Kanellopoulos [45] have also reported a comparable reduction in spherical equivalent and cylindrical error in both accelerated and conventional CXL.

Results on UCVA and BVCA reported in the literature are conflicting for these techniques, with either no change or an improvement reported following CXL. Wittig-Silva [56] and Coskunseven et al. [57] showed in their studies an increase in UCVA and BCVA (*p* < 0.01) compared to controls. Ng et al. [52] showed no significant difference in the change of BCVA at 12 months after CXL in both conventional and accelerated CXL groups. Only UCVA showed mild but significant improvement of 0.13 lines. In our study, UCVA increased in the same manner in the conventional group and the accelerated CXL group, as showed by the higher improvement at years 1 and 2. However, the improvement became comparable from the first year after the procedure. Our findings revealed that BCVA in the conventional treated group had a higher improvement at 1 year compared to the accelerated one but stabilized thereafter. Thus, at 3, 4, and 5 years following the procedure, patients in the accelerated group presented an improved BCVA compared to the conventional group (*p* < 0.05 for both time points). The results in BCVA improvement in the conventional group are similar with those we published before [19].

In our study, we evaluated the HOA and RMS parameters, and we demonstrated a statistically significant reduction in both groups compared with baseline. Similarly, Greenstein et al. [58] revealed a significant decrease in HOA, total coma, three-order coma, and vertical coma. Moreover, Caporossi et al. [14] showed a significant decline of HOA and coma aberration as early and long-term results (2 years), following CXL. Kirgiz et al. [32] demonstrated a real improvement in coma values in the accelerated CXL with 10 minutes at 1-year follow-up.

Our findings showed a statistically significant reduction in the topographical index (TP), corneal volume, ISV, IHA, IHT, BAD_D, and ART Max from baseline, but there was no significant difference between the two groups (*p* < 0.05 at all time points). Similarly, Kirgiz et al. [32] revealed the same results regarding the above parameters.

Demarcation line was limited to the anterior-mid stroma until there was a maximum depth of 202 ± 12.03 *μ*m in the accelerated group when compared with conventional CXL that reached 214 ± 12.82 *μ*m.

Doors showed similar results [38] and described the best visibility of the corneal demarcation line using AS-OCT at 1 month after CXL treatment with an average of depth of 313 *μ*m. Wollensak et al. [11] described cellular apoptosis to a depth of 300 *μ*m radiating with UVA at 3 mW/cm^2^. Furthermore, similar results were demonstrated by Seiler and Hafezi [59], Kymionis et al. [60], Doors et al. [38], and Yam et al. [36]. In accelerated CXL, Seiler and Hafezi [59], Moramarco et al. [37], and Kymionis et al. [60] demonstrated a demarcation line depth of 213–215 *μ*m after 1 month following the procedure. Similar to our observation, Kimionis et al. [60] noted that a 10-minute treatment with 9 mW/cm^2^ resulted in a demarcation line that was less deep (288.46 ± 42.37 *μ*m) compared to the standard procedure (350.75 ± 49.34 *μ*m). They also reported that a modified accelerated protocol of CXL (9 mw/cm^2^ for 14 minutes) provides the same demarcation line depth as the conventional procedure (294.38 ± 60.57 *μ*m in the accelerated group and 380.78 ± 54.99 *μ*m in the conventional group). The difference was not statistically significant. Kymionis [60] and Mazzota et al. [61] demonstrated a deep demarcation line of 280 *μ*m in the pulsed-light cross-linking procedure applied for 6 minutes.

This paper has several limitations that should be acknowledged. This was a retrospective study, and there was no randomization. However, the strength of our paper comes from the relatively large sample size compared to previous reports and on the long-term follow-up of these patients, adding to previous knowledge on this subject. To our knowledge, this is the first comparative study done in Romania and is the first one with such a long period of follow-up (5 years).

## 5. Conclusion

Our study revealed the efficacy and safety of long-term follow-up (5 years) in accelerated CXL in comparison with the conventional protocol. Following both protocols, a stabilization of KCN after 1 year was obtained.

## Figures and Tables

**Table 1:** Gender, age, and keratoconus stage in both groups.

Parameter	Conventional CXL (S-CXL) *N* = 93 eyes (93 patients)	Accelerated CXL (A-CXL) *N* = 76 eyes (76 patients)	*p* value
Men, *n* (%)	52 (55.9)	41 (53.9)	0.184
Mean age, years	26.5 ± 8.5	29 ± 8.2	0.064

Keratoconus stage, *n* (%)			
I	8 (8.6)	6 (7.89)	<0.001
II	50 (53.76)	36 (47.37)	
III	35 (37.63)	34 (44.74)	

**Table 2:** Evolution of Kflat, Ksteep, Kavg, and Kmax in both groups.

	Kflat			Ksteep			Kavg			Kmax		
	Conventional CXL	Accelerated CXL	*p* value	Conventional CXL	Accelerated CXL	*p* value	Conventional CXL	Accelerated CXL	*p* value	Conventional CXL	Accelerated CXL	*p* value
Preop	47.47 ± 4.15	46.67 ± 4.34	0.293	50.42 ± 4.87	49.98 ± 4.51	0.098	48.95 ± 4.16	48.33 ± 4.14	0.309	54.05 ± 5.41	56.07 ± 5.39	0.179
1 year	46.45 ± 3.94	46.15 ± 4.45	0.465	49.54 ± 4.71	49.22 ± 4.11	0.709	48.00 ± 4.06	47.69 ± 4.36	0.522	52.1 ± 5.35	54.45 ± 5.42	0.06
2 years	46.4 ± 4.00	46.1 ± 4.40	0. 672	49.45 ± 4.81	49.08 ± 4.61	0.455	47.93 ± 4.10	47.59 ± 4.32	0.387	51.99 ± 5.35	54.22 ± 5.44	0.09
3 years	46.39 ± 3.99	45.98 ± 4.36	0.198	49.43 ± 4.81	48.99 ± 4.56	0.43	47.91 ± 4.10	47.49 ± 4.33	0.6	51.78 ± 5.28	54.02 ± 5.38	0.126
4 years	46.37 ± 3.71	45.93 ± 4.38	0.32	49.42 ± 4.90	48.91 ± 4.54	0.57	47.90 ± 4.30	47.42 ± 4.30	0.299	51.74 ± 5.31	54.01 ± 5.38	0.113
5 years	46.29 ± 3.69D	45.88 ± 4.39D	0.864	49.40 ± 4.88D	48.88 ± 4.54D	0.494	47.89 ± 4.30	47.40 ± 4.30	0.247	51.72 ± 5.32	54 ± 5.39	0.114

**Table 3:** Topographical parameter evolution in both groups.

Parameter		S-CXL	*p* value compared to baseline	A-CXL	*p* value compared to baseline	*p* value between groups
TP	Preoperative	462.419 ± 34.36		463.565 ± 35.53		0.832
	1 year	445.698 ± 38.90	0.0022	450.052 ± 33.34	0.0168	0.4416
	2 years	444.043 ± 38.98	0.0008	447.223 ± 33.25	0.0039	0.574
	3 years	442.677 ± 38.9	0.0003	441.527 ± 41.7	0.0006	0.8532
	4 years	441.419 ± 38.97	0.0001	441.263 ± 42.02	0.0006	0.9863
	5 years	441.15 ± 38.9	0.0001	441.132 ± 42.23	0.0006	0.9518
Vol C	Preoperative	57.452 ± 6.55		57.069 ± 3.46		0.6463
	1 year	56.173 ± 6.55	0.018	55.803 ± 3.28	0.0221	0.6547
	2 years	56.136 ± 6.53	0.0179	55.464 ± 3.27	0.0038	0.4151
	3 years	56.103 ± 6.55	0.0162	55.064 ± 3.63	0.0007	0.2182
	4 years	56.104 ± 6.55	0.0162	55.059 ± 3.64	0.0006	0.2179
	5 years	56.093 ± 6.56	0.0159	55.058 ± 3.64	0.0006	0.2236
IVA	Preoperative	0.900 ± 0.41		1.001 ± 0.32		0.0825
	1 year	0.760 ± 0.4	0.0199	0.851 ± 0.32	0.005	0.1149
	2 years	0.752 ± 0.39	0.0123	0.843 ± 0.32	0.0028	0.0851
	3 years	0.743 ± 0.41	<0.0001	0.832 ± 0.34	0.0015	0.1131
	4 years	0.735 ± 0.4	<0.0001	0.821 ± 0.34	0.0008	0.115
	5 years	0.730 ± 0.41	<0.0001	0.815 ± 0.32	0.0004	0.217
ISV	Preoperative	79.354 ± 29.53		89.578 ± 27.53		0.222
	1 year	75.569 ± 29.57	0.0383	84.078 ± 26.84	0.0214	0.0541
	2 years	74.58 ± 29.55	0.0271	82.165 ± 26.22	0.0091	0.0892
	3 years	73.741 ± 29.58	0.0197	81.066 ± 26.67	0.0055	0.0964
	4 years	73.661 ± 29.59	0.0154	80.889 ± 26.44	0.0558	0.0731
	5 years	73.466 ± 29.6	0.0124	80.678 ± 26.84	0.0558	0.057
IHA	Preoperative	30.817 ± 21.3		34.315 ± 23.59		0.3141
	1 year	27.439 ± 20.57	0.0262	29.074 ± 20.15	0.0215	0.8265
	2 years	27.083 ± 20.41	0.0223	28.774 ± 21.25	0.0219	0.7823
	3 years	26.801 ± 20.35	0.019	28.362 ± 20.63	0.0103	0.6216
	4 years	26.553 ± 20.3	0.0164	28.023 ± 21.02	0.0101	0.6782
	5 years	26.315 ± 20.28	0.0141	27.974 ± 21.22	0.0101	0.7348
BAD_D	Preoperative	7.806 ± 3.15		8.663 ± 2.15		0.455
	1 year	7.593 ± 3.14	0.0445	8.220 ± 2.22	0.0397	0.1372
	2 years	7.548 ± 3.15	0.0057	8.170 ± 2.08	0.0352	0.1409
	3 years	7.51 ± 3.15	0.0152	8.150 ± 2.19	0.0164	0.13
	4 years	7.481 ± 3.15	0.0148	8.109 ± 2.38	0.0134	0.1136
	5 years	7.462 ± 3.18	0.0045	8.048 ± 2.07	0.0088	0.1064
ART Max	Preoperative	175.021 ± 67.32		169.881 ± 55.36		0.594
	1 year	163.892 ± 66.99	0.2599	152.052 ± 48.59	0.0365	0.1994
	2 years	161.924 ± 66.99	0.1852	148.644 ± 48.68	0.0131	0.1506
	3 years	160.451 ± 67.09	0.141	146.292 ± 48.34	0.0026	0.0763
	4 years	159.344 ± 67.07	0.1133	145.287 ± 48.08	0.0026	0.0918
	5 years	158.86 ± 67.12	0.1028	144.092 ± 48.12	0.0026	0.1093

**Table 4:** Abberometric parameter evolution in both groups.

Parameter		S-CXL	*p* value compared to baseline	A-CXL	*p* value compared to baseline	*p* value between groups
HOA	Preoperative	8.383 ± 3.81		8.962 ± 2.93		0.2785
	1 year	7.745 ± 3.65	0.0244	8.311 ± 3.16	0.018	0.28
	2 years	7.733 ± 3.64	0.0236	8.224 ± 3.43	0.0122	0.3617
	3 years	7.719 ± 3.65	0.0226	8.164 ± 3.38	0.0099	0.3992
	4 years	7.709 ± 3.65	0.0219	7.998 ± 3.21	0.0086	0.3881
	5 years	7.706 ± 3.65	0.0217	7.989 ± 3.03	0.0067	0.3151
RMS	Preoperative	193.823 ± 16.63		195.142 ± 14.1		0.584
	1 year	187.370 ± 16.78	0.0092	193.120 ± 13.75	0.0372	0.175
	2 years	186.374 ± 16.73	0.0027	192.820 ± 13.52	0.0305	0.177
	3 years	185.373 ± 16.68	0.0007	192.670 ± 13.74	0.0275	0.269
	4 years	185.186 ± 16.71	0.0005	192.446 ± 13.66	0.0206	0.22
	5 years	185.075 ± 16.73	0.0004	192.388 ± 13.7	0.019	0.118

## Data Availability

The data used and analyzed in the present study are available from the corresponding author.
